# Effect of Methamphetamine on Spectral Binding, Ligand Docking and Metabolism of Anti-HIV Drugs with CYP3A4

**DOI:** 10.1371/journal.pone.0146529

**Published:** 2016-01-07

**Authors:** Anantha R. Nookala, Junhao Li, Anusha Ande, Lei Wang, Naveen K. Vaidya, Weihua Li, Santosh Kumar, Anil Kumar

**Affiliations:** 1 Division of Pharmacology & Toxicology, School of Pharmacy, University of Missouri Kansas City, Kansas City, Missouri, United States of America; 2 Shanghai key laboratory of new drug design, School of Pharmacy, East China University of Science and Technology, Shanghai, China; 3 Department of Mathematics and Statistics, University of Missouri Kansas City, Kansas City, Missouri, United States of America; 4 Department of Pharmaceutical Sciences, College of pharmacy, University of Tennessee Health Science Center, Memphis, Tennessee, United States of America; University of Rochester Medical Center, UNITED STATES

## Abstract

Cytochrome P450 3A4 (CYP3A4) is the major drug metabolic enzyme, and is involved in the metabolism of antiretroviral drugs, especially protease inhibitors (PIs). This study was undertaken to examine the effect of methamphetamine on the binding and metabolism of PIs with CYP3A4. We showed that methamphetamine exhibits a type I spectral change upon binding to CYP3A4 with δA_max_ and K_D_ of 0.016±0.001 and 204±18 μM, respectively. Methamphetamine-CYP3A4 docking showed that methamphetamine binds to the heme of CYP3A4 in two modes, both leading to N-demethylation. We then studied the effect of methamphetamine binding on PIs with CYP3A4. Our results showed that methamphetamine alters spectral binding of nelfinavir but not the other type I PIs (lopinavir, atazanavir, tipranavir). The change in spectral binding for nelfinavir was observed at both δA_max_ (0.004±0.0003 vs. 0.0068±0.0001) and K_D_ (1.42±0.36 vs.2.93±0.08 μM) levels. We further tested effect of methamphetamine on binding of 2 type II PIs; ritonavir and indinavir. Our results showed that methamphetamine alters the ritonavir binding to CYP3A4 by decreasing both the δA_max_ (0.0038±0.0003 vs. 0.0055±0.0003) and K_D_ (0.043±0.0001 vs. 0.065±0.001 nM), while indinavir showed only reduced K_D_ in presence of methamphetamine (0.086±0.01 vs. 0.174±0.03 nM). Furthermore, LC-MS/MS studies in high CYP3A4 human liver microsomes showed a decrease in the formation of hydroxy ritonavir in the presence of methamphetamine. Finally, CYP3A4 docking with lopinavir and ritonavir in the absence and presence of methamphetamine showed that methamphetamine alters the docking of ritonavir, which is consistent with the results obtained from spectral binding and metabolism studies. Overall, our results demonstrated differential effects of methamphetamine on the binding and metabolism of PIs with CYP3A4. These findings have clinical implication in terms of drug dose adjustment of antiretroviral medication, especially with ritonavir-boosted antiretroviral therapy, in HIV-1-infected individuals who abuse methamphetamine.

## Introduction

Cytochrome P450 (CYP) belongs to the family of heme proteins that are involved in the biotransformation of xenobiotics. Among the various CYP isozymes, CYP3A4 metabolizes approximately 50% of the currently marketed drugs including several antiretroviral drugs [1]. Most antiretroviral drugs including protease inhibitors (PIs) and non-nucleoside reverse transcriptase inhibitors (NNRTIs) are substrates, inducers and/or inhibitors of CYP3A4 [2]. The highly active antiretroviral therapy (HAART) regimens contain multiple drugs including PIs and NNRTIs, which also causes potential drug-drug interactions through CYP3A4 [3, 4]. Among all the PIs, ritonavir is known to be the strongest inhibitor of CYP3A4 and is, therefore, used as a booster in the HAART regimen that contains NNRTIs and PIs. Based on the physicochemical properties of the PIs, several studies have shown that PIs exhibit two types of spectral changes upon binding with CYP3A4 known as type I and type II [5, 6].

Methamphetamine is a commonly used substance of abuse among HIV-1 infected population. Studies have shown that the risk of acquiring HIV-1 infection is higher among men who have sex with men (MSM) and abuse amphetamines compared to those MSM who don’t abuse drugs [7, 8]. Furthermore, several *in vitro* and *in vivo* studies have shown the role of methamphetamine in altering HIV-1 pathogenesis by various mechanisms, including suppression of innate restriction factors in macrophages and increased viral loads in the brain [9, 10]. Few reports have shown the occurrence of fatal interactions in patients abusing methamphetamine while on ritonavir therapy resulting from inhibition of CYP2D6 mediated methamphetamine metabolism [11]. However, it’s unclear whether methamphetamine also reduces the response to HAART by altering the bioavailability of HAART and increasing HAART-mediated toxicity that would ultimately result in increased HIV-1 pathogenesis in methamphetamine users. Since, methamphetamine is also partly metabolized by CYP3A4, which is known to metabolize NNRTIs and PIs, we propose that methamphetamine interacts with NNRTIs/PIs through CYP3A4. Therefore, in this study we determined the interaction of methamphetamine with CYP3A4 followed by the effect of methamphetamine on the interaction of PIs with CYP3A4 using spectral binding and docking studies.

## Materials and Methods

### Materials

Plasmid encoding CYP3A4 was generously provided by Dr. James Halpert (Skaggs School of pharmacy and pharmaceutical sciences, UCSD). Ni-NTA agarose column was obtained from Qiagen (Valencia, CA). Methamphetamine was purchased from Sigma chemicals (St.Louis, MO, USA). All the protease inhibitors (PIs) were obtained from NIH AIDS reagent center. XTerra ^®^ MS C18 column (4.6X50mm, i.d, 3.5 μm) was purchased from Waters (Milford, MA, USA). The other reagents used in the study were obtained from standard commercial sources.

### Enzyme preparation

Histidine tagged CYP3A4 enzyme was expressed in E. *coli* and purified on Ni-NTA agarose column as described previously [12]. Briefly, CYP3A4 cDNA was introduced into TOPP3 strain of E. *coli* by transformation and the cells were plated on LB ampicillin plate with tetracycline. The next day, a viable and isolated colony was grown in TB media for 48 h to induce CYP3A4. The cultures were harvested by centrifugation and CYP3A4 was extracted from the membrane using detergent followed by ultracentrifugation. The supernatant was loaded on a Ni-NTA agarose column for further purification. The purified enzyme was dialyzed and final preparation was stored at -80^ο^C until use.

### Spectral binding assay

The spectral change was recorded by using a UV/Vis double beam spectrophotometer (6800 UV/VIS JENWAY spectrophotometer) as described earlier [6]. Briefly, 0.5 μM of purified CYP3A4 enzyme was incubated with 2 ml of 0.1 M HEPES buffer for 3 min and distributed equally between the sample and reference cuvettes. Depending upon the solvent used to solubilize methamphetamine or PIs, equal quantities of either methanol or water was added into the reference cuvette with final volume of the external solvent <1%. Increasing concentrations of methamphetamine was added to the sample cuvette while the same volume of solvent was added into the reference cuvette and the spectral change was recorded. For experiments involving PIs, different concentrations of PIs were added into the sample cuvette and the spectral change was recorded with a 2 min interval between each addition. PIs binding to CYP3A4 exhibit two different types of spectral change based on the presence of different kinds of functional groups. Type I ligands displace the heme water ligand, thus shifting the iron spin equilibrium towards the high-spin form resulting in a spectrum, which is characterized by an increase in the absorbance at 390 nm (high spin) and a decrease at 420 nm (low spin). Type II ligands containing nitrogen heterocycles replace the water ligand to stabilize the low-spin form resulting in a spectrum that is characterized by an increase in the spectrum at ~430 nm and a decrease at ~400 nm. The effect of methamphetamine on the spectral binding of PIs was determined by incubating the CYP3A4 enzyme with 100 μM of methamphetamine and titrating with increasing concentrations of PIs.

### PIs Metabolism Assay

The effect of Methamphetamine on CYP3A4 mediated metabolism of PIs was assessed using human liver microsomes (HLMs) (Invitrogen, MA, USA), which possess high CYP3A4 activity. Briefly, the metabolite formations were assessed in incubation mixtures (0.5 mL) containing 100 mM phosphate buffer (pH 7.4), HLMs (0.1 mg/mL), and 5 mM Mgcl_2_ [13, 14]. The incubation mixtures were pre-incubated with six different concentrations of Meth ranging from 3 μM to 1000 μM for 10 min, followed by the addition of 5 μl stock solution of PI. The entire mixture was incubated in water bath at 37°C for 15 min. The substrate concentrations of the PI’s used were near their K_m_ values; ritonavir (2 μM), lopinavir (10 μM), and nelfinavir M-8 (1 μM). Later, the reactions were initiated upon the addition of 100 μl of 25 mM NADPH (final concentration of 5 mM) solution to each of the tube and incubated in water bath at 37°C for 60 min. After the incubation period, 500 μl of Stop solution (Acetonitrile: water: FA in 47:50:3 ratio) containing 0.7 μM of internal standard (IS), Atazanavir-d5), was added in each tube. Then, the tubes were vortexed and centrifuged at 500 g for 10 min and supernatant was transferred into vials for analysis by LC-MS/MS.

### Analytical instrumentation

**Fig 1:**
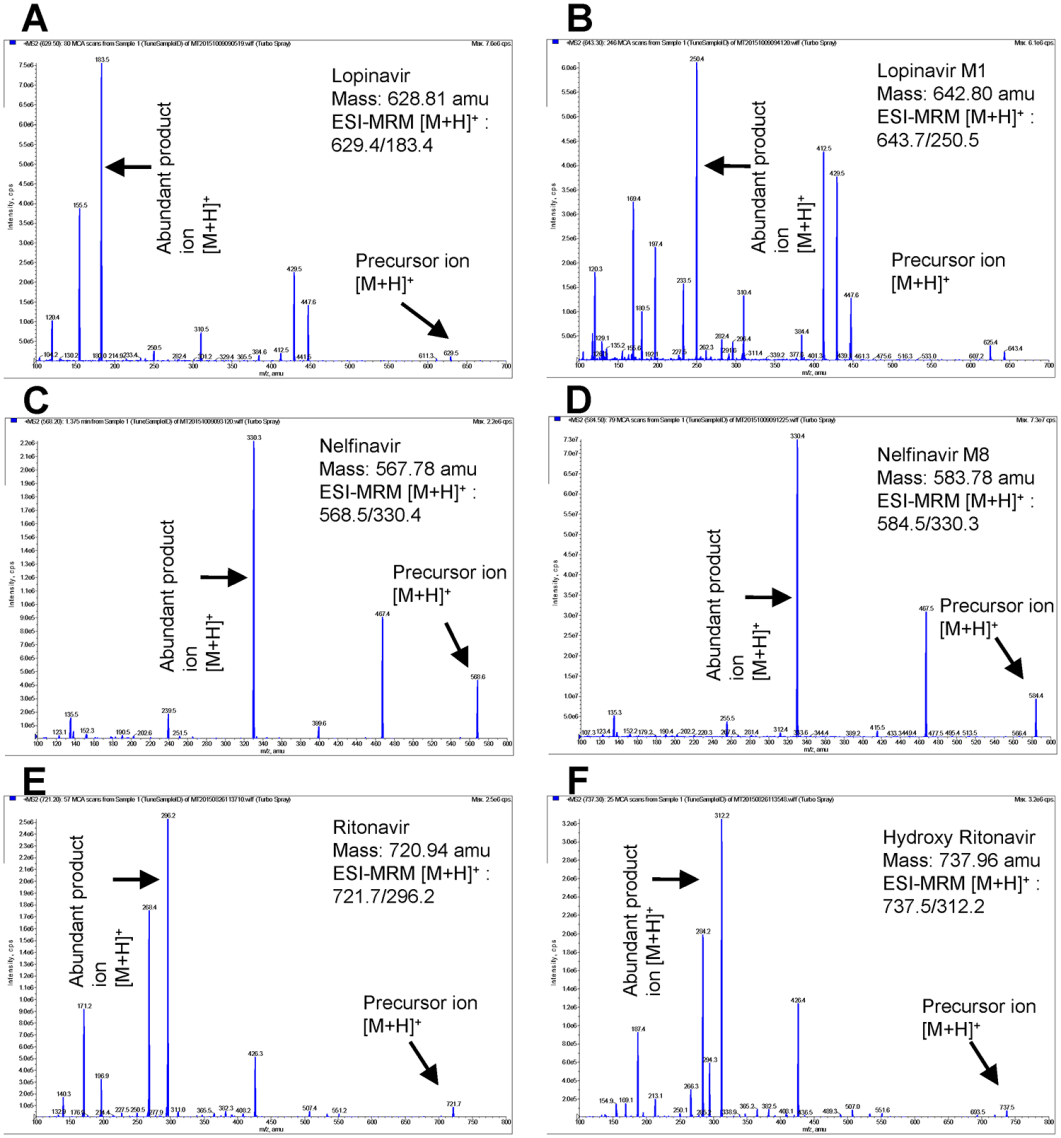
Development of LC-MS/MS methods to quantitate lopinavir, lopinavir M1, nelfinavir, nelfinavir M8, ritonavir, and hydroxy ritonavir in human liver microsomes. MS/MS spectra of (A) lopinavir, (B) lopinavir M1, (C) nelfinavir, (D) nelfinavir M8, (E) ritonavir, (F) hydroxy ritonavir with ESI proton adducts [M+H]^+^ in positive mode. The y-axis shows intensity (CPS, count per second) and the x-axis shows the mass to charge ratio (m/z, amu).

Ritonavir, lopinavir, lopinavir M1, nelfinavir, nelfinavir M8, and atazanvir d5 stock solutions were prepared in methanol where as hydroxy ritonavir was prepared in DMSO. Atazanavir d5 was used as an IS for all the analytes. Standard curves for each analyte with IS were generated in blank HLMs incubation mixture as described above. The carry over test was performed by injecting an extra blank following the injection of upper limit of quantitation (ULOQ) of the standard curve with an IS. The LC-MS/MS methods were developed for each analyte along with its metabolite using 3200 QTRAP LC-MS/MS system, AB Sciex. The most suitable multiple reaction monitoring (MRM) transitions produced through electron spray ionization [M+H]^+^ for precursor ions (Q1) and product ions (Q3) were found to be 721.2→296.2 for ritonavir, 737.5→312.2 for hydroxy ritonavir, 629.4→183.4 for lopinavir, 643.7→250.5 for lopinavir M1, 568.5→330.4 for nelfinavir, 584.5→330.3 for nelfinavir M8, and 710.3→340.5 for atazanavir d5. (Fig 1, Table 1). A source temperature of 500°C, ion spray voltage of 5500 V, curtain gas of 20, GS 1 & 2 of 50, and dwell time of 200 msec were used for the determination of all analytes. The MRM method parameters such as declustering potential (DP), entrance potential (EP), collision energy (CE), and collision cell exit potential (CXP), were listed in the Table 1 along with the MRM transitions.

**Table 1:** Summary of optimized parameters and characteristics for positive mode LC—MS/MS detection of Protease inhibitors.

	MRM Parameters						
Name	Q1	Q3	DP (V)	EP (V)	CE (V)	CXP (V)	t_r_ (min)
**Lopinavir**	629.3	183.3	32	6	30	6	6.64
**Lopinavir M1**	643.3	250.3	32	6	37	6	6.36
**Nelfinavir**	568.6	330.4	70	7	50	5	5.42
**Nelfinavir M8**	584.6	330.4	70	7	50	5	4.49
**Ritonavir**	721.2	312.2	58	7	25	5	6.41
**Hydroxy ritonavir**	737.2	340.5	58	7	25	5	5.26

All the analytes were separated on reverse phase XTerra ^®^ MS C18 column (4.6X50mm, i.d, 3.5 μm) using a Shimadzu LC-20AD HPLC system (CA, USA). A binary gradient elution method comprising of mobile phases (acetonitrile with 0.1% formic acid and water with 0.1% formic acid) with a flow rate of 0.5 mL/min was employed. For separation of ritonavir, hydroxy ritonavir, lopinavir, and lopinavir M1, a gradient program starting with 35% acetonitrile with 0.1% formic acid for the first minute, followed by a linear gradient to 100% acetonitrile with 0.1% formic acid in 5 min, maintained for 1 min and then to 35% acetonitrile with 0.1% formic in 4 min with a total run time of 11 min was developed. Under the above conditions, ritonavir, hydroxy ritonavir, lopinavir, lopinavir M1, and IS exhibited retention times of 6.41, 5.26, 6.64, 6.36, and 4.33 min, respectively. For separation of nelfinavir and nelfinavir M8, the same gradient profile was used except for starting with 35% of 70% acetonitrile with 0.1% formic acid. Upon using the above method, nelfinavir, nelfinavir M8, and IS were eluted in 5.42, 4.49, and 6.33 min, respectively. Data acquisition and analyses were performed using the Analyst 1.4.2 software package (Applied Biosystems, Foster City, CA, USA).

### Docking studies

The study was performed as described previously with modifications [15]. The initial CYP3A4 model was taken from Protein Data Bank (PDB, www.pdb.org). Currently, there are 17 crystal structures of human CYP3A4 available in PDB. On the basis of crystal resolution, residues completeness, and ligand size, 3NXU (at 2.00 Å resolution) was chosen. There are 2 asymmetric molecules in 3NXU. Chain A was used for docking simulations. The missing residues were reconstructed using 1TQN and 3V0M as the templates. Docking of PIs into the CYP3A4 active site in the absence and presence of methamphetamine was accomplished by Gold 5.2 software [16]. The center of the grid was placed on the centroid of ritonavir, a co-crystalized ligand in 3NXU. The residues within 20 Å of the ligand were defined as the binding pocket. Chemscore was used for scoring the interactions between the ligands and CYP3A4. The output solution was set 30 for each run. The first 10 poses were analyzed in detailed.

### Statistical analysis

The K_D_ and δA_max_ were determined by fitting the curve (absorbance vs. concentration) with hyperbolic equation using non-regression analysis in sigma plot 11 (Systat Software, San Jose, CA). The p-values were determined using One way ANOVA with Dunnet’s post-hoc test and values <0.05 were considered statistically significant.

## Results and Discussion

### Methamphetamine-CYP3A4 spectral binding

**Fig 2:**
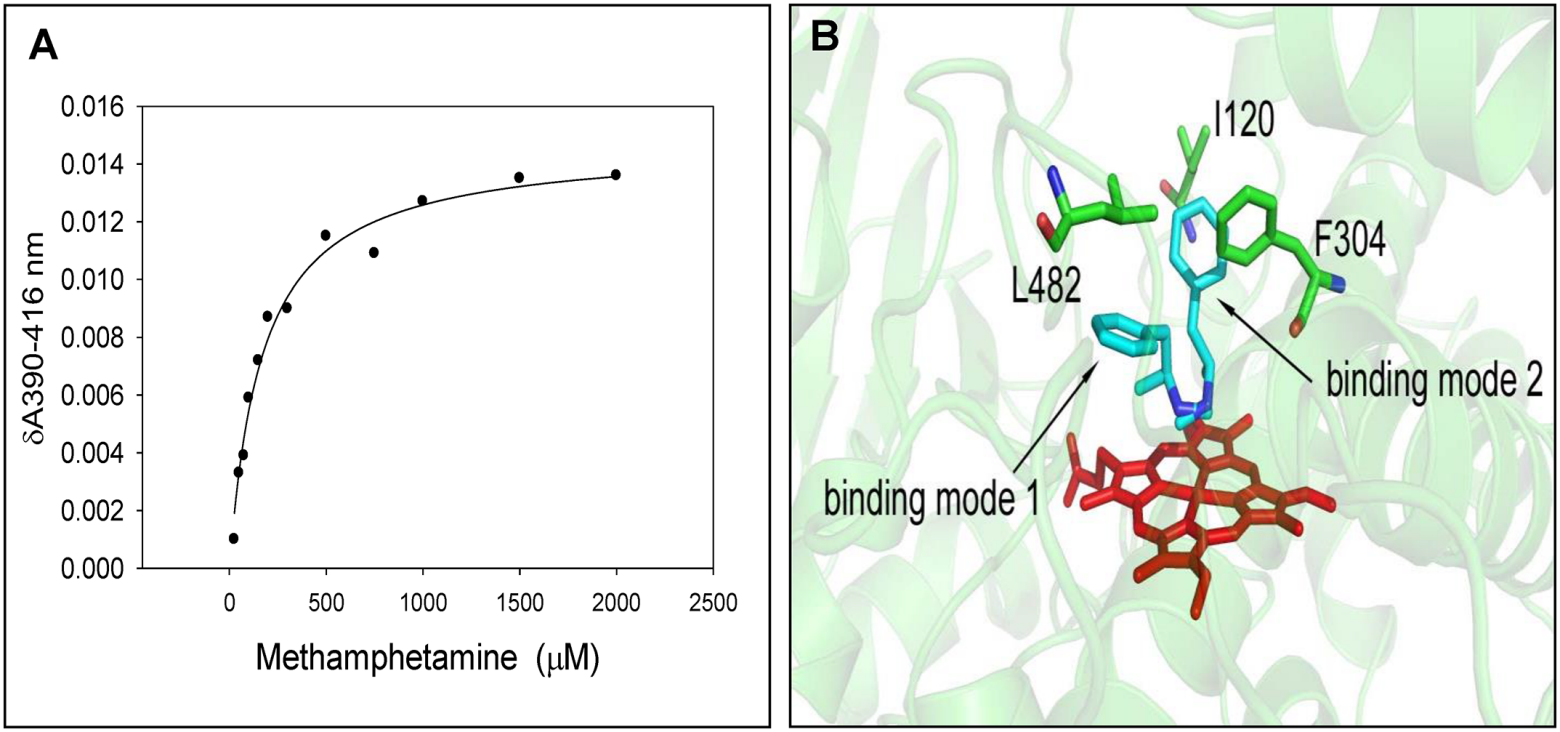
Spectral binding of methamphetamine with CYP3A4. (A) The spectral binding was performed at varying concentrations of methamphetamine from 1 μM to 2 mM. The graph was plotted using difference in absorbance at 390 and 416 nm vs. concentration of methamphetamine. The graph was fitted with hyperbolic equation using non-linear regression analysis in sigma plot 11. (B) Docking simulation of methamphetamine binding with CYP3A4 in two different binding modes. The heme, methamphetamine, and interacting amino acid residues of CYP3A4 are represented in red, green, and blue, respectively.

The current study was undertaken to examine the role of methamphetamine on the spectral binding of PIs with CYP3A4. In order to determine this, first we examined the spectral binding of methamphetamine with CYP3A4. The results showed an increase in absorbance at 390 nm and a decrease at 418 nm, suggesting that methamphetamine shows a type I spectral change with CYP3A4. The result is consistent with its physicochemical properties as it does not contain any reactive group with a loan pair, e.g. imidazole, which usually shows type II spectral change through covalent binding. The titration of methamphetamine with CYP3A4 showed a hyperbolic spectral binding (Fig 2A). Therefore, we fitted the methamphetamine-CYP3A4 binding using hyperbolic equation, which yielded a K_D_ of methamphetamine as 204 ± 18 μM and δA_max_ at 0.016 ± 0.001. The result from δA_max_ suggests that methamphetamine causes approximately 20% spectral transition (low to high spin) with CYP3A4 at saturating concentration of methamphetamine. This spectral transition with CYP3A4 is similar to spectral transition obtained from other type I ligands such as ethanol [17]. To determine and validate the nature of methamphetamine binding with CYP3A4, we performed methamphetamine docking with CYP3A4. The results show that methamphetamine interact with CYP3A4 active site in two different binding modes, both of which can undergo N-demethylation (Fig 2B).

The involvement of various CYP enzymes including CYP3A4 in the metabolism of methamphetamine has been shown in previous studies, including ours [18–22]. Our docking data is consistent with methamphetamine CYP3A4 interaction that prefers N-demethylation route of metabolism. Further, spectral binding of methamphetamine with CYP3A4 suggests that methamphetamine-CYP3A4 interaction can be studied using type I spectral change. It is important to mention that all the ligands, which are either substrates, inhibitors, or activators of CYP3A4, do not necessarily show spectral change. If a ligand does not show spectral change with CYP3A4, then it is very difficult to study ligand-CYP3A4 interaction and its effect on the metabolism, inhibition, or activation of other CYP3A4 ligands. Although the K_D_ obtained from spectral binding is higher than the plasma concentration of methamphetamine (2–10 μM) [23], it is likely that its concentration in the liver is higher than in plasma because methamphetamine is rapidly metabolized in the liver. Further, the methamphetamine level observed in the post-mortem brains of chronic abusers ranges between 0.8 mM to 1mM [24]. CYP3A4 is known to be present in different brain cells [25], and in some cells, the local CYP3A4 concentration is as high as the liver CYP3A4. Thus, CYP3A4-methamphetamine interaction at such concentration is of significance in the brain. Recent study has shown that methamphetamine mediates blood brain barrier (BBB) dysfunction leading to internalization of occludin [26]. A leaky BBB may also increase the transport of other compounds including its own, as well as, NNRTIs and PIs in the brain. Thus, in addition to liver, potential drug-drug interactions between methamphetamine and NNRTIs/PIs through CYP3A4 may also occur in the brain.

Since CYP3A4 is involved in the metabolism of both PIs and NNRTIs, it is important to study drug-drug interactions in the presence of methamphetamine [27]. We and others have earlier characterized the binding of CYP3A4 with PIs and classified PIs-CYP3A4 interactions into type I PIs, type II PIs, and unbound PIs (PIs that do not show spectral change) [5, 6]. Furthermore, we have shown that ethanol differentially alters the binding of both type I and type II PIs with CYP3A4, suggesting a three-way ethanol-CYP3A4-PI interaction [6, 17]. In this study, we have studied the effect of methamphetamine on the spectral binding of both type I and type II PIs.

### Effect of methamphetamine on interaction of Type I PIs with CYP3A4

**Fig 3:**
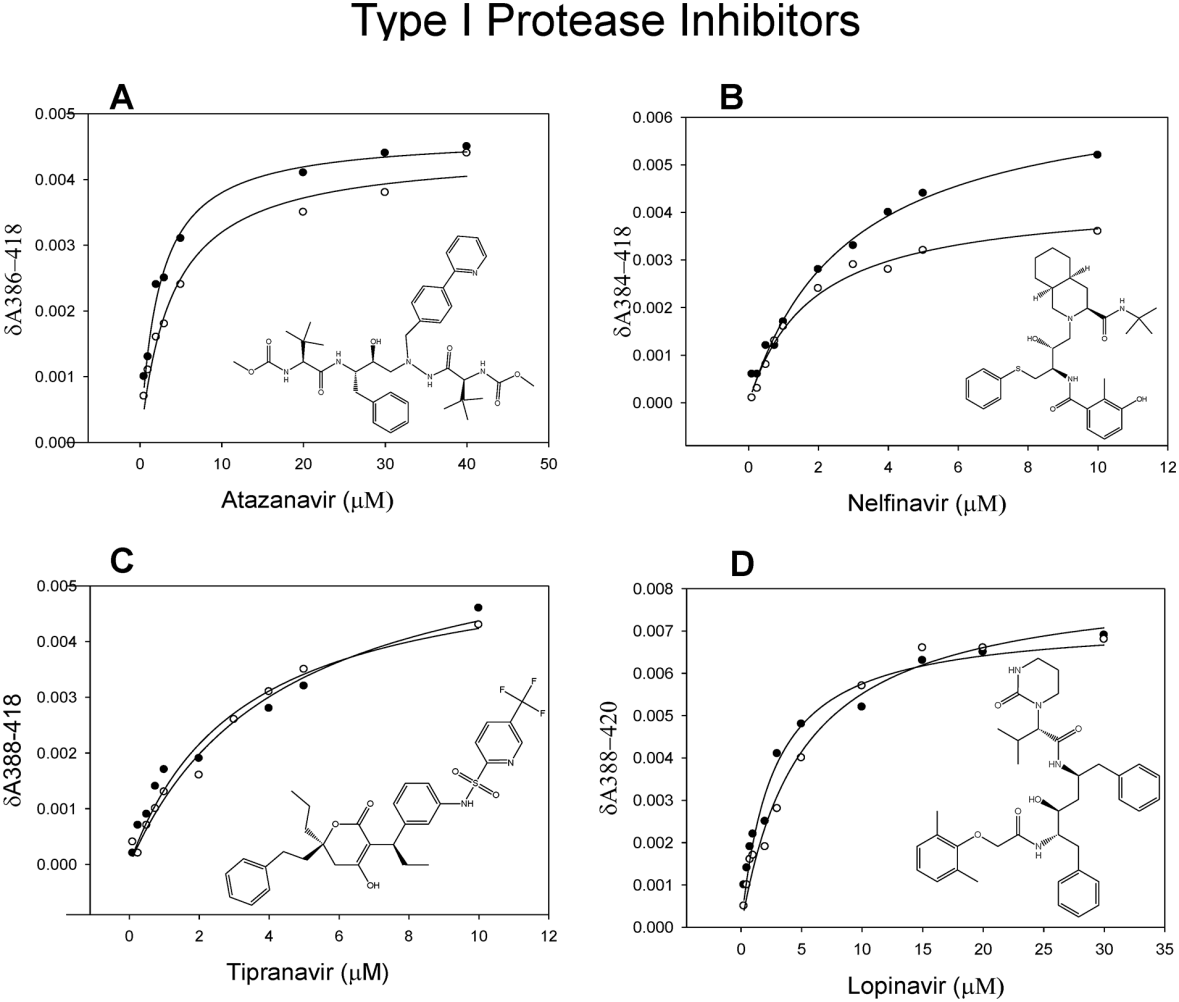
Spectral binding of type I PIs with CYP3A4 in the absence (filled circles) and presence (open circles) of methamphetamine. (A-D) The spectral binding was performed at varying concentrations of atazanavir, nelfinavir, tipranavir and lopinavir. The K_D_ and δA_max_ for each PI with and without methamphetamine are presented in Table 2. One way ANOVA with Dunnet’s post-hoc test was employed to calculate the statistical significance. A p-value <0.05 is indicated by * and <0.1 is indicated by #.

**Fig 4:**
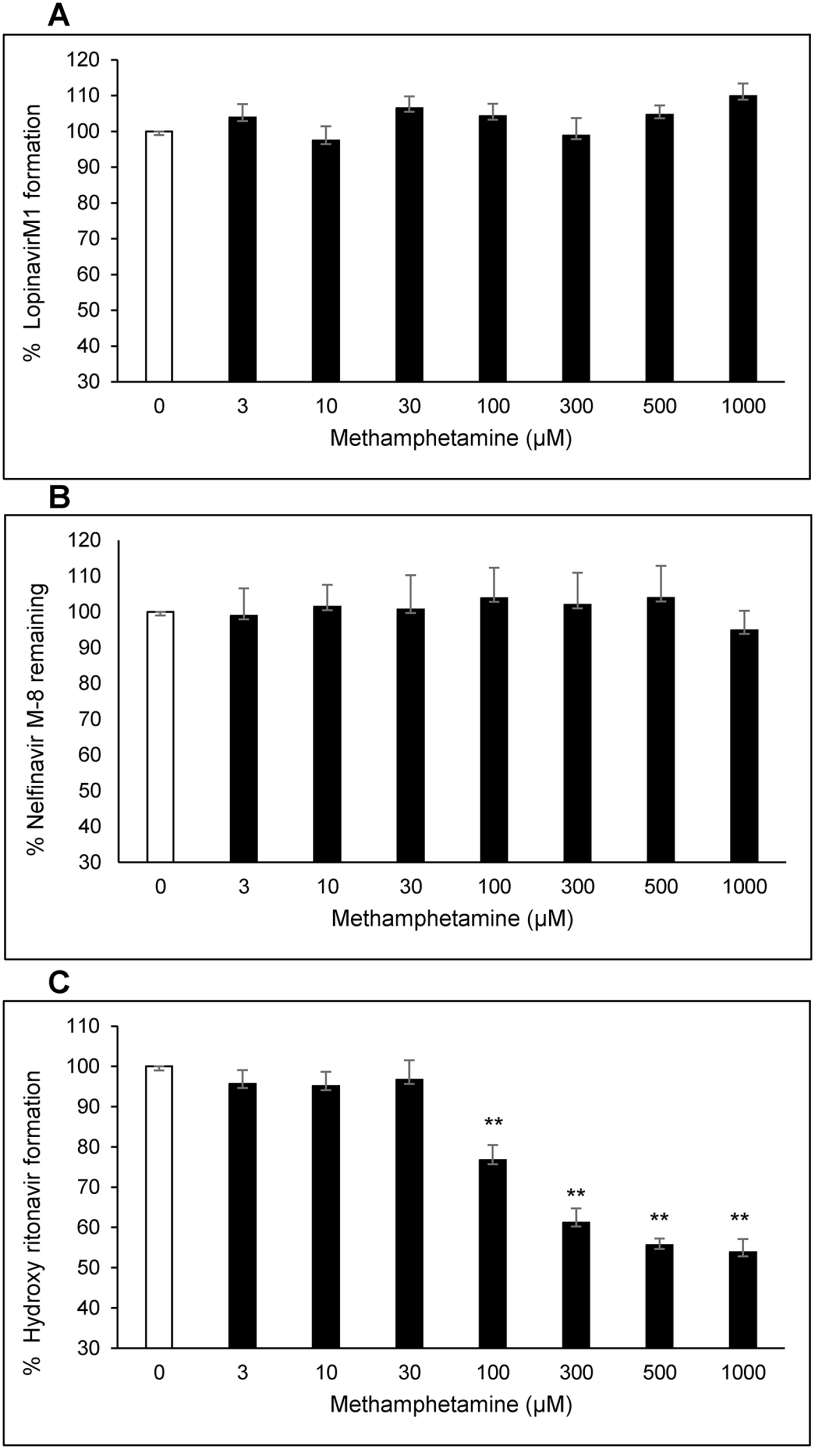
Effect of methamphetamine on metabolism of PIs in CYP3A4 human liver microsomes. Effect of methamphetamine on (A) lopinavir M1 formation from lopinavir, (B) nelfinavir M8 degradation and, (C) hydroxy ritonavir formation from ritonavir. One way ANOVA with Dunnet’s post-hoc test was employed to calculate the statistical significance and ** denotes p-value <0.01.

We examined the role of methamphetamine on spectral binding of four type I PIs (atazanavir, nelfinavir, tipranavir and lopinavir) with CYP3A4. Our results showed that methamphetamine causes a significant decrease in the δA_max_ of nelfinavir (0.004 ± 0.0003 vs. 0.0068 ± 0.0001) and a slight increase in δA_max_ with tipranavir (0.0061 ± 0.0002 vs. 0.0050 ± 0.0005). However, it did not show any change in δA_max_ with atazanavir and lopinavir (Fig 3, Table 2). Further, methamphetamine slightly increased the K_D_ values of lopinavir, atazanavir, and tipranavir, while it significantly decreased the K_D_ of nelfinavir (1.42 ± 0.36 vs. 2.93 ± 0.08 μM) (Table 2). To determine the effect of methamphetamine on CYP3A4-mediated metabolism of these PIs (whose metabolites are commercially available), we performed inhibition studies using HLMs. Inhibition studies were performed by analyzing the rate of metabolite formation of lopinavir M1 from lopinavir and decrease in the substrate concentration of nelfinavir M8 in the presence of varying concentrations of methamphetamine. Our studies showed that methamphetamine did not affect the metabolism of either lopinavir or nelfinavir M8 as shown in the Fig 4A and 4B. The results are more or less similar to the results obtained by CYP3A4-PI binding experiments.

**Table 2:** The K_D_ and δA_max_ of all the PIs with and without methamphetamine.

No Methamphetamine				100 μM Methamphetamine	
	PIs	δAmax	KD (μM)	δAmax	KD (μM)
	Lopinavir	0.0070 ± 0.0002	2.5 ± 0.1	0.0078 ± 0.0005	4.1 ± 0.8
**Type I**	Atazanavir	0.0045 ± 0.0002	3.2 ± 0.8	0.0046 ± 0.0002	4.7 ± 0.7
	Tipranavir	0.0050 ± 0.0005	3.1 ± 0.1	0.0061 ± 0.0002	4.7 ± 0.4
	Nelfinavir	0.0068 ± 0.0001	2.9 ± 0.1	0.0040 ± 0.0003*	1.4 ± 0.3^#^
**Type II**	Ritonavir	0.0055 ± 0.0003	0.065 ± 0.001	0.0038 ± 0.0003*	0.043 ± 0.001*
	Indinavir	0.0044 ± 0.0002	0.174 ± 0.030	0.0039 ± 0.0002	0.086 ± 0.010*

*-indicates a p-value <0.05;

^#^-Indicates p-value <0.1

In general, methamphetamine did not significantly alter the binding of Type I PIs with CYP3A4, except for nelfinavir. In consistence with the spectral findings, our inhibition studies in microsomal system did not show any significant effect of methamphetamine on type I PIs metabolism.

**Fig 5:**
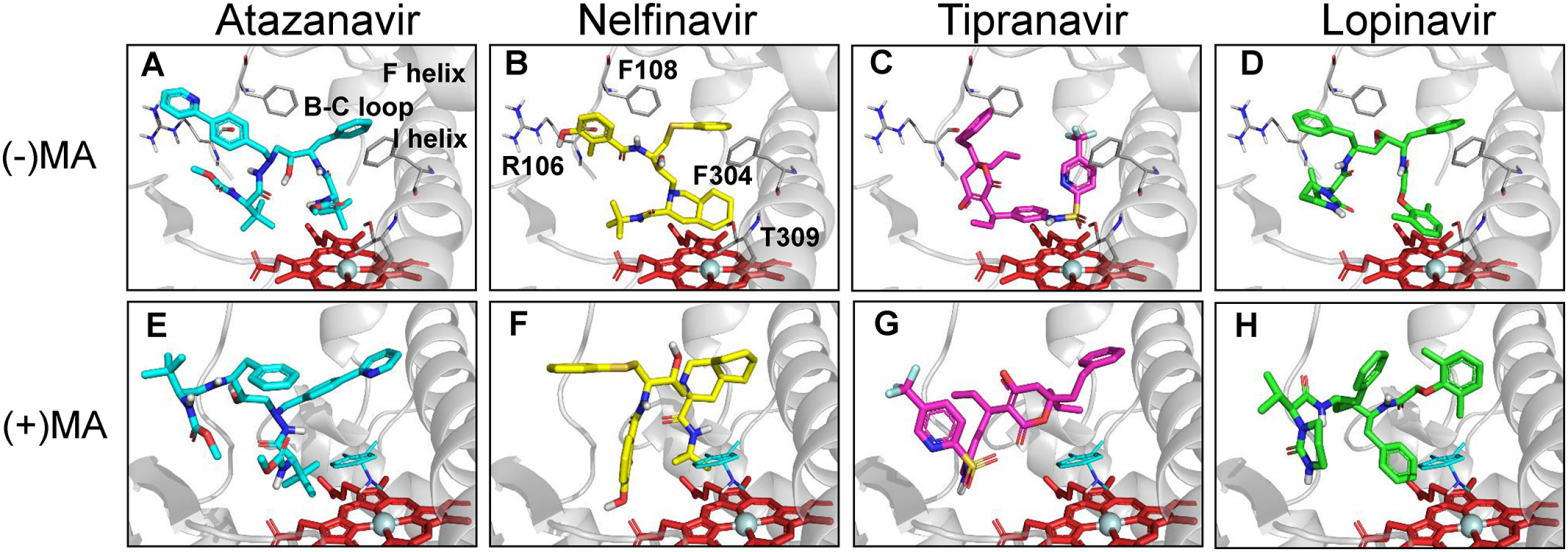
Docking simulations of PIs in the absence and presence of methamphetamine. Structures of (A) Atazanavir, (B) Nelfinavir, (C) Tipranavir and (D) Lopinavir with their preferred binding sites pointing towards the heme moiety of CYP3A4in the absence of methamphetamine. Docking simulations of (E) Atazanavir, (F) Nelfinavir, (G) Tipranavir and (H) Lopinavir with heme moiety of CYP3A4 in the presence of methamphetamine. R-methamphetamine in binding mode 1 was shown in cyan sticks. Docking was performed as described in Materials and Methods.

To corroborate these findings, we performed docking studies with Type I PIs in the absence and presence of methamphetamine. Docking of PIs in the active site of CYP3A4 in the absence of methamphetamine showed binding modes that could be classified into 2–3 clusters in the top ten poses for each PI. The predicted binding a region with the modes of the 4 PIs occupied boundaries being closer to I helix, B-C loop, K-L loop, and C-terminal loop (Fig 5A). Average docking scores in the major cluster (cluster with highest average docking score) for type I PIs ranged from 44.62 to 51.60 (Table 3). The docking scores of different PIs were in general consistent with the relative ranking of the experimental K_D_ values. Since the crystal structure of CYP3A4 complexed with MA is not available, we first predicted the possible binding modes of MA in the active site of CYP3A4. Our docking studies suggested two possible binding modes for both R-methamphetamine and S-methamphetamine (Fig 2B). The docking scores of Type I PIs in the presence of R-methamphetamine in relatively more preferred binding mode 1 did not show significant differences in average scores compared to that in the absence of methamphetamine (Table 3). These results are in agreement with binding and metabolism studies in which we saw no significant effect of methamphetamine. Even though there is slight difference in the average scores with S-methamphetamine in both binding modes, we do not think these values make a significant difference in terms of metabolism (data not shown).

**Table 3:** The statistical results of PIs docking into CYP3A4 active site in the presence of methamphetamine.

	R-MA mode1	Average score ^[^^a^^]^	Average score ^[^^b^^]^	ΔScore	Average distance (Å) ^[^^c^^]^	Average distance (Å) ^[^^d^^]^	ΔDistance (Å)
	Lopinavir	51.6	47.84	3.76	3.16	5.45	-2.29
**Type I**	Atazanavir	44.62	41.88	2.74	3.61	6.42	-2.81
	Tipranavir	46.09	46.9	-0.81	3.75	6.33	-2.58
	Nelfinavir	49.16	47.57	1.59	3.6	5.31	-1.71
**Type II**	Ritonavir	57.56	46.75	10.81	3.16	5.7	-2.54
	Indinavir	55.88	52.37	3.51	3.37	5.74	-2.37

^**a**^. Average score of major cluster in the absence of methamphetamine

^**b**^. Average score of major cluster in the presence of methamphetamine

^**c**^. The average distance between the atom of site closest to Fe and Fe for all top 10 conformers in the absence of methamphetamine.

^**d**^. The average distance between the atom of site closest to Fe and Fe for all top 10 conformers in the presence of methamphetamine.

Using molecular modeling it is difficult to predict which binding mode or which molecule is more stable, because CYPs exhibit considerable structural flexibility [28, 29]. This has been manifested by our docking simulations that no significant differences between the docking scores have been observed.

Overall, our data suggests that methamphetamine does not alter the spectral change (δA_max_ and K_D_), inhibition of CYP3A4, and substrate docking of type I PIs with CYP3A4s. Together these results suggest that there is no major conformational change in the active site of CYP3A4 when type I PIs bind with CYP34 in the presence of methamphetamine. Overall, the findings from type I PIs suggest no significant drug-drug interaction between methamphetamine and type I PIs. This is in contrast with our previous study with ethanol-CYP3A4 interaction, in which, ethanol showed altered metabolism of nelfinavir [17]. Therefore, a drug-dose adjustment with the type I PIs when given alone may not be needed. However, further *ex vivo* study is also needed to test whether the metabolism, bioavailability, and efficacy of type I PIs is altered in methamphetamine users.

### Effect of methamphetamine on interaction of Type II PIs with CYP3A4

**Fig 6:**
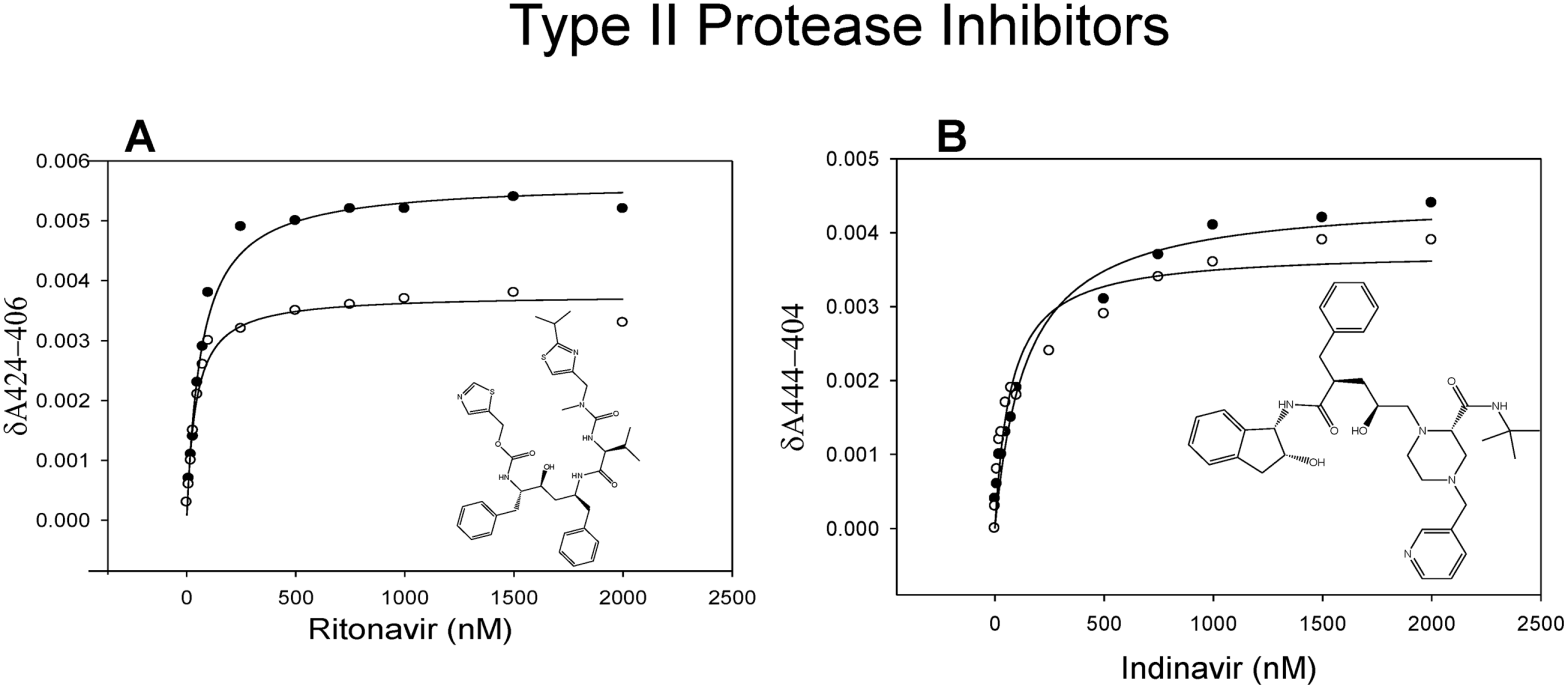
Spectral binding of type II PIs with CYP3A4 in the absence (filled circles) and presence (open circles) of methamphetamine. (A, B) The spectral binding was performed at varying concentrations of ritonavir and indinavir. The K_D_ and δA_max_ for each PI with and without methamphetamine are presented in Table 2. One way ANOVA with Dunnet’s post-hoc test was employed to calculate the statistical significance. A p-value <0.05 is indicated by * and <0.1 is indicated by #.

We also examined the effect of methamphetamine on spectral binding of two type II PIs (ritonavir and indinavir) with CYP3A4. Methamphetamine significantly decreased the δA_max_ of ritonavir (0.0038 ± 0.0003 vs. 0.0055 ± 0.0003) but didn’t affect indinavir (0.0039 ± 0.0002 vs. 0.0044 ± 0.0002) (Fig 6, Table 2). However, methamphetamine significantly decreased the K_D_ values of both ritonavir and indinavir by approximately 33% and 50%, respectively (0.043 ± 0.0001 vs. 0.065 ± 0.001 for ritonavir and 0.086 ± 0.01 vs. 0.174 ± 0.03 μM for indinavir) (Table 2). We also assessed the formation of hydroxy ritonavir, one of the major metabolites of ritonavir through CYP3A4 pathway, in the presence of varying concentrations of methamphetamine [30]. Our results demonstrated a significant decrease in the formation of hydroxy ritonavir in CYP3A4 microsomes with increasing concentrations of methamphetamine. As shown in Fig 4C, 100 μM of methamphetamine significantly decreased the formation of hydroxy ritonavir (23.3 ± 3.8%) with a further decrease in the metabolite level as the methamphetamine concentration increased to 1000 μM (46.2 ± 3.3%).

**Fig 7:**
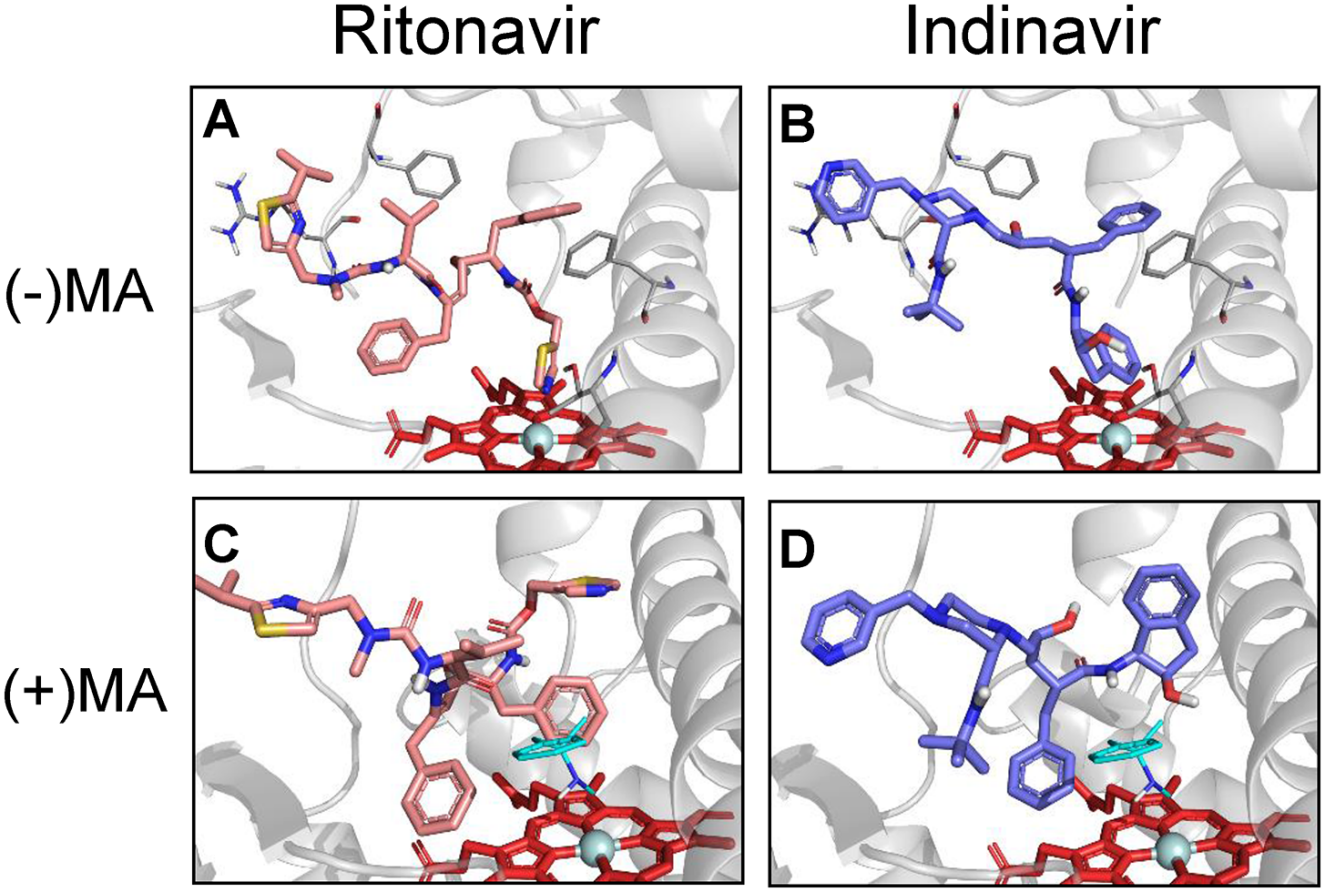
Docking simulations of Type II PIs in the absence and presence of methamphetamine. Structures of (A) Ritonavir and (B) Indinavir with their preferred binding sites pointing towards the heme moiety of CYP3A4in the absence of methamphetamine. Docking simulations of (C) Ritonavir and (D) Indinavir with heme moiety of CYP3A4 in the presence of methamphetamine. R-methamphetamine in binding mode 1 was shown in cyan sticks. Docking was performed as described in Materials and Methods.

Further, we performed ritonavir docking to CYP3A4 to verify whether methamphetamine alters binding mode of ritonavir. The docking study showed that ritonavir has three sites that can interact with the heme of CYP3A4 (Fig 7A). However, the thiazole site of ritonavir is the most prominent because it exhibits 6 conformers with CYP3A4 with the highest score of 66.92, and the distance between ritonavir and heme is the shortest (2.75 Å). Interestingly, in the presence of methamphetamine, ritonavir exhibits altered binding with CYP3A4, in which thiazole group of ritonavir points away from the heme. Similarly, the docking scores of ritonavir binding are lower (50.33 vs. 66.92) and cluster are higher (10 vs. 3) in the presence of methamphetamine than in the absence of methamphetamine (S1 Table). Indinavir docking into CYP3A4 showed a slight decrease in the average score of major cluster in the presence of methamphetamine both binding modes (Table 3).

Ritonavir exhibited the lowest K_D_ among all the drugs studied, which is consistent with the previous finding [6], and with the finding that it is the most potent inhibitor of CYP3A4 [30]. Most importantly, methamphetamine further increased the binding affinity of both the type II PIs with CYP3A4. It is possible that methamphetamine causes a conformational change in CYP3A4 active site that enables the binding of type II PIs with CYP3A4 relatively more strongly. On the other hand, methamphetamine decreased the magnitudes of binding of ritonavir with CYP3A4 as determined by a decrease in δA_max_ and a decrease in ritonavir metabolism. These findings suggest that methamphetamine acquires selective binding with certain pool of CYP3A4, which can’t be replaced by ritonavir, a strongest CYP3A4 inhibitor known. Furthermore, docking studies suggest that thiazole group of ritonavir moves away from the heme site of CYP3A4 in the presence of methamphetamine suggesting a decrease in the magnitude of binding. Thus, the docking study is consistent with the finding that the δA_max_ decreases in the presence of methamphetamine and also with decreased metabolite formation. Similarly, an increased binding affinity with ritonavir (relative to lopinavir) in the presence of methamphetamine can be explained by a higher magnitude of decreased binding energy and increased clusters with ritonavir compared to lopinavir (S1 Table). It is likely that methamphetamine binding causes a conformation change in the active site of CYP3A4 that leads to increased affinity with ritonavir and indinavir.

Overall, our findings suggest that methamphetamine alters type II PIs-CYP3A4 interactions, which lead to decreased metabolism of these PIs, especially ritonavir. The results have clinical implications because an altered interaction of type II PIs, especially ritonavir, is expected to alter the inhibition characteristics of ritonavir and thus the metabolism of other PIs or NNRTIs. An increased metabolism of type II PIs is expected to decrease their bioavailability and therefore decrease the response of HAART. In addition, an increased metabolism may cause increased accumulation of their metabolites leading to increased toxicity. On the other hand since ritonavir is used as an inhibitor of CYP3A4 to increase the bioavailability of other PIs and NNRTIs, an increased inhibition may further increase the bioavailability of other drugs. Although this may cause an improved response to HAART, this may also cause increased drug-mediated toxicity. Therefore, there is a need for drug dose adjustment in methamphetamine users who are on ritonavir-boosted PIs or NNRTIs in their antiretroviral regimen. Further studies are needed to examine the metabolism, bioavailability, and efficacy of these drugs in the presence of methamphetamine in HIV-1-infected monocytes and lymphocytes, as well as, using ex-vivo study in HIV-infected methamphetamine users who are on HIV medication.

## Supporting Information

S1 Table The difference in docking scores and clusters of CYP3A4 bound with ligands between with and without the presence of methamphetamine (MA). (DOC)
