# Supplementary material for: Effect of Methamphetamine on Spectral Binding, Ligand Docking and Metabolism of Anti-HIV Drugs with CYP3A4
Source: PLoS One. 2016 Jan 7;11(1):e0146529. doi: 10.1371/journal.pone.0146529 (PMC4704828; doi:10.1371/journal.pone.0146529)
Supplement: S1 Table — (DOC) [file pone.0146529.s001.doc]

**Effect of methamphetamine on spectral binding, ligand docking and metabolism of anti-HIV drugs with CYP3A4**

Anantha R Nookala^1¶^, Junhao Li^2¶^, Anusha Ande^1^, Lei Wang^2^, Naveen K Vaidya^3^, Weihua Li^2^, Santosh Kumar^4^ and Anil Kumar^1*^

^1^Division of Pharmacology & Toxicology, School of Pharmacy, University of Missouri Kansas City, Kansas city, Missouri, United States of America

^2^Shanghai key laboratory of new drug design, School of Pharmacy, East China University of Science and Technology, Shanghai, China

^3^Department of Mathematics and Statistics, University of Missouri Kansas City, Kansas city, Missouri, United States of America

^4^Department of Pharmaceutical Sciences, College of pharmacy, University of Tennessee Health Science Center, Memphis, Tennessee, United States of America

*Corresponding author

Email: [kumaran@umkc.edu](mailto:kumaran@umkc.edu) (AK)

^¶^These authors equally contributed to this work

**S1 Table:** **The difference in docking scores and clusters of CYP3A4 bound with ligands between with and without the presence of methamphetamine (MA).**

|  | Highest score | | Clusters | |
| --- | --- | --- | --- | --- |
|  | Ritonavir | Lopinavir | Ritonavir | Lopinavir |
| No MA | 66.92 | 54.06 | 3 | 3 |
| MA: Mode I | 50.33 | 44.28 | 10 | 7 |
| Difference (Δ) | 16.59 | 9.78 | 7 | 4 |
| MA: Mode II | 43.95 | 43.64 | 10 | 10 |
| Difference (Δ) | 22.97 | 10.42 | 7 | 7 |

Difference (Δ) corresponds to the differences in highest scores or clusters between MA and without MA for ritonavir and lopinavir in both the binding modes. These differences are much higher with ritonavir than lopinavir.
